# Profiling of the Microbiome Associated With Nitrogen Removal During Vermifiltration of Wastewater From a Commercial Dairy

**DOI:** 10.3389/fmicb.2018.01964

**Published:** 2018-08-20

**Authors:** Ellen Lai, Matthias Hess, Frank M. Mitloehner

**Affiliations:** ^1^Department of Animal Science, University of California, Davis, Davis, CA, United States; ^2^DOE Joint Genome Institute, Walnut Creek, CA, United States

**Keywords:** 16S rRNA, dairy wastewater, microbiome, nitrogen cycling, vermifiltration

## Abstract

Vermifiltration is a biological treatment process during which earthworms (e.g., *Eisenia fetida*) and microorganisms reduce the organic load of wastewater. To infer microbial pathways responsible for nutrient conversion, past studies characterized the microbiota in vermifilters and suggested that nitrifying and denitrifying bacteria play a significant role during this wastewater treatment process. In contrast to previous studies, which were limited by low-resolution sequencing methods, the work presented here utilized next generation sequencing to survey in greater detail the microbiota of wastewater from a commercial dairy during various stages of vermifiltration. To complement sequence analysis, nitrogenous compounds in and gaseous emissions from the wastewater were measured. Analysis of 16S rRNA gene profiles from untreated wastewater, vermifilter influent, and vermifilter effluent suggested that members of *Comamonadaceae*, a family of the *Betaproteobacteria* involved in denitrification, increased in abundance during the vermifiltration process. Subsequent functional gene analysis indicated an increased abundance of nitrification genes in the effluent and suggested that the nitrogen removal during vermifiltration is due to the microbial conversion of ammonia, a finding that was also supported by the water chemistry and emission data. This study demonstrates that microbial communities are the main drivers behind reducing the nitrogen load of dairy wastewater during vermifiltration, providing a valuable knowledge framework for more sustainable and economical wastewater management strategies for commercial dairies.

## Introduction

Intensification of agriculture has accentuated the need for efficient waste management strategies to reduce nitrogen (N) loading in wastewater. Proper waste management not only mitigates the organic load at the point source, but also prevents downstream environmental and public health issues related to N that leaches into soil and groundwater, which can result in eutrophication, soil acidification, and groundwater pollution (Galloway et al., 2003). To address the need for effective wastewater treatment systems for agricultural systems and rural areas, vermifiltration has been suggested before as a decentralized, low-cost, and low-maintenance wastewater treatment solution (Aguilera, 2003).

During vermifiltration, organic wastewater is applied to a bed of organic media (e.g., woodchips, sawdust, straw) seeded with epigeic earthworms (e.g., *Eisenia fetida* and *Eisenia andrei*), and organic matter is subsequently degraded through the symbiotic activities of earthworms and microorganisms (Zhao et al., 2010; Li et al., 2013). Past studies suggest that earthworms contribute indirectly to nutrient removal by facilitating microbial growth (Zhao et al., 2010; Liu et al., 2013; Wang et al., 2016). Earthworms mechanically degrade organic input and excrete mucus and casts, respectively enriched in organic matter, nitrogen, and phosphorus, making these nutrients readily available to microorganisms (Aira et al., 2007; Zhao et al., 2010). In addition to increasing nutrient availability through their excretions, earthworms create spatially separate aerobic and anaerobic microenvironments, supporting the growth of both aerobic and anaerobic microorganisms (Wang et al., 2011a). Aerobic microenvironments originate from earthworms’ tunneling activity, which increases the porosity of the media, aerates the media, and in turn facilitates the growth of aerobic microorganisms (Zhao et al., 2010; Wang et al., 2011a). The anaerobic microenvironment of the earthworm gut, on the other hand, stimulates anaerobes that are otherwise dormant in the aerobic external environment as earthworms ingest the media (Horn et al., 2003).

The combined effect of increased nutrient availability and the creation of dual microenvironments favors a distinct microbial community in vermifilters. Previous studies used polymerase chain reaction-denaturing gradient gel electrophoresis (PCR-DGGE) to analyze the biofilm that formed on the media during laboratory-scale vermifiltration of domestic wastewater. These studies suggested that the presence of earthworms results in higher abundance of *Alpha-, Beta*-, and *Gammaproteobacteria* within the biofilm (Wang et al., 2011b; Liu et al., 2012; Li et al., 2013). Similarly, Liu and colleagues observed that *Proteobacteria* dominated the biofilm that formed in a vermifilter during the treatment of rural domestic sewage (Liu et al., 2013). Taxonomic groups found to be enriched in the biofilm included bacteria that drive conversions of carbon and nitrogen, such as *Betaproteobacteria, Gammaproteobacteria*, ammonia-oxidizing bacteria, and *Nitrospira* (Wang et al., 2011b; Xing et al., 2012; Liu et al., 2013; Wang and Chu, 2016).

To evaluate the N cycling potential of microbial communities in wastewater treatment, previous studies quantified the abundance of marker genes involved in N transformations as a proxy for the microbial nitrogen transformation. More specifically, *amoA*, which encodes the active site of ammonia monooxygenase and catalyzes the conversion of ammonia (NH_3_) to nitrite (NO_2_^-^), was quantified via qPCR from swine wastewater lagoons (Ducey et al., 2011), constructed wetlands (Paranychianakis et al., 2016), and lab-scale biofilters operated without worms (Ji et al., 2013). Similarly, *nirS, nirK*, and *nosZ* were quantified to estimate the activity of denitrifiers in anaerobic swine manure and during lab-scale biofiltration in the presence and absence of earthworms (Ducey et al., 2011; Ji et al., 2013; Wang and Chu, 2016). In addition, DGGE (Wang et al., 2011a) and sequencing (Ducey et al., 2011) was utilized to estimate the diversity of these key genes. Although these early studies provided first insights into the metabolic activity of complex microbial systems (i.e., wastewater rich in organic material (Ducey et al., 2011; Wang and Chu, 2016) and soil (Petersen et al., 2012) they were limited by the low resolution or high costs of the approaches utilized (Muyzer et al., 1993; Muyzer and Smalla, 1998). With the sequencing technologies and bioinformatics tools that have become available over the last years, targeted metatranscriptomics combined with metagenomics analyzes to generate highly specific reference database have set the stage for more conclusive studies in this area. These shotgun approaches will also address amplification biases and reference database limitations that are some of the major limitations that have been encountered in previous studies.

The objective of this study was to determine if 16S rRNA gene-based sequencing can be utilized successfully to (i) characterize the microbial community in the different stages of a commercial scale vermifilter that processes dairy wastewater and (ii) provide a foundation for optimizing the microbial community to maximize N removal from dairy wastewater. Hence, we tested the simple hypothesis that the differences in community composition from the untreated wastewater and from the effluent water exiting the vermifilter would reflect an enhanced abundance of organisms associated with N-cycling function as well as an enhanced inferred capability of N-related biochemistry within the effluent.

To this end and to overcome the limitations associated with the previous studies, we generated more holistic microbial community profiles associated with different stages of the vermifiltration process by targeting the 16S rRNA gene and predicting the N-cycling potential of these microbial communities PICRUSt, (Langille et al., 2013). To validate the *in-silico* prediction of the N-cycling potential, predicted nitrogen transformations were compared to N profiles of the wastewater and of the gases emitted from the wastewater and the vermifilter.

## Materials and Methods

### Experimental Site

**FIGURE 1:**
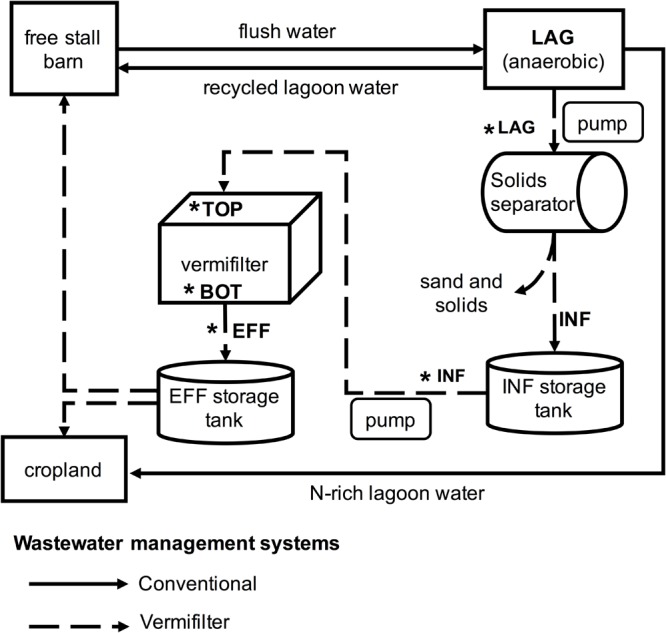
Overview of conventional and vermifiltration wastewater management systems of the present study. In the conventional system of the dairy for the present study, the free stall barn is flushed 2 or 3 times a day, and the flush water is sent to the anaerobic lagoon. The lagoon water (LAG) is then recycled back to the free stalls as flush water, or applied to cropland. In the vermifiltration system, LAG is pumped through a rotary screen to remove sands and solids, and the resulting liquid, the influent (INF), is stored in the storage tank. For the first 15 min of every hour, the INF is sprinkled over the top of the vermifilter (TOP). The INF percolates to the bottom of the vermifilter (BOT) via gravity, and the resulting effluent (EFF) is stored in a storage tank until it is used as flush water or applied to cropland. Asterisks indicate wastewater collection sites and vermifilter sampling locations for gas measurements.

This study was conducted at a commercial dairy located in the California San Joaquin Valley that houses a total of 1,300 cows, including 760 milking cows. The free-stall barn was flushed 3 times daily for 6 min using recycled wastewater from an open wastewater lagoon with a holding capacity of ∼5.7 million L. Lagoon water (LAG) was pumped into a rotary screen solids separator (Biofiltro, Fresno, CA, United States) equipped with a 200 mm screen to remove sand and other solids. The resulting influent water (INF) was then directed into the top opening of a holding tank until ∼2,100 L had been collected, a process that took ∼7 min. Every hour, the entire 2,100 L of freshly generated INF was applied for 10 min to the vermifilter’s surface (measuring 49 m × 11 m) using a rotary head sprinkler system. The applied INF percolated to the bottom of the filtration system where the resulting effluent water (EFF) exited the vermifiltration system. EFF was stored until it was recycled as flush water or applied as fertilizer to adjacent cropland. An overview of the vermifiltration wastewater management system and a comparison to the conventional wastewater management system is provided in **Figure 1**. The vermifilter consisted of a concrete pool (49 m × 11 m × 1.5 m) filled with a 30 cm bottom layer of river cobbles (∼10 cm × 20 cm) to improve drainage, topped with a 1.2 m layer of semi-sterile woodchips made from hearts of Douglas fir, White fir, and Ponderosa pine. The surface layer (∼30 cm) of woodchips was seeded with 300 kg earthworms (*Eisenia fetida*). Twenty peripheral PVC exhaust pipes (20 cm diameter) allowed air exchange between the bottom layer and ambient air (**Supplementary Figure S1**). Temperature of the lagoon water (24.9°C), influent water (26.3°C), vermifilter surface layer (20.4°C) and effluent water (25.8°C) were measured when samples for DNA extraction were collected.

### Sampling

Wastewater samples were collected in triplicates during October 2015. At each site (LAG, INF, and EFF), three 45 mL wastewater samples were collected using sterile conical tubes. LAG was collected from an outlet on the solids separator discharging lagoon water. INF was collected from a spigot between the influent holding tank and the pipe leading to the vermifilter. EFF was collected from the pipe draining the effluent into the effluent holding tank. All nine samples were immediately stored at -20°C until DNA extraction.

### DNA Extraction

Samples were thawed in a 25°C water bath and centrifuged at 2,190 × *g* for 15 min at 4°C (Sovall RT6000B, DuPont Co. Wilmington, DE, United States). DNA extraction was performed after centrifugation using the FastDNA SPIN Kit for Soil (MP Biomedicals, Solon, OH, United States) with ∼500 mg pellet following a modified version of the manufacturer’s protocol. Specifically, centrifugation times were increased until sufficient pelleting was observed (i.e., supernatant was clear). Additionally, the incubation time during the DNA binding to the matrix was extended to 1 h to increase DNA recovery. Extracted DNA was stored at -20°C until subsequent PCR amplification was performed.

### PCR Amplification, Library Preparation, and Sequencing

The V4 region of the 16S rRNA gene was amplified using primers 515F and 806R (Caporaso et al., 2012). For sequencing, forward and reverse sequencing oligonucleotides were designed to contain a unique 8 nt barcode (N), a primer pad (underlined), a linker sequence (italicized), and the Illumina adaptor sequences (bold). Each sample was barcoded with a unique forward and reverse barcode combination. (Forward primer construct: **AATGATACGGCGACCACCGAGATCTACAC-**NNNNNNNN-TATGGTAATT-*GT-*GTGCCAGCMGCCGCGGTAA; reverse primer construct: **CAAGCAGAAGACGGCATACGAGAT**NNNNNNNNAGTCAGTCAG*CC*GGACTACHVGGGTWTCTAAT.) Barcode combinations for each sample are provided in **Supplementary Table S1**. Each PCR reaction contained 1 Unit Kapa2G Robust Hot Start Polymerase (Kapa Biosystems, Boston, MA, United States), 1.5 mM MgCl_2_, 10 pmol of each primer, and 1μL of DNA. The PCR was performed using the following conditions: 95°C for 2 min, followed by 30 cycles of 95°C for 10 s, 55°C for 15 s, 72°C for 15 s and a final extension of 72°C for 3 min. The amplicon was quantified using a Qubit instrument with the Qubit High Sensitivity DNA kit (Invitrogen, Carlsbad, CA, United States). Individual amplicon libraries were pooled, cleaned with Ampure XP beads (Beckman Coulter, Brea, CA, United States), and sequenced using 250 bp paired-end sequencing on an Illumina MiSeq instrument at the DNA Technologies Core in the University of California, Davis Genome Center. Raw sequence reads were submitted to NCBI’s Sequence Read Archive and deposited under the accession numbers: SAMN06909136, SAMN06909137, SAMN06909138, SAMN06909139, SAMN06909140, SAMN06909141, SAMN06909142, SAMN06909143, and SAMN06909144.

### Sequence Analysis

Sequencing resulted in a total of 188,870 raw reads, which were analyzed using mothur v1.37.4 (Schloss et al., 2009) and the MiSeq standard operating procedure accessed on 05/11/2016 (Kozich et al., 2013). Using the *make.contig* command, raw sequences were combined into contigs, which were filtered using *screen.seqs* to remove sequences > 275 bp or that contained ambiguous base calls to reduce PCR and sequencing error. Duplicate sequences were merged with *unique.seqs*, and the resulting unique sequences were aligned to the V4 region of the SILVA SEED alignment reference v123 (Quast et al., 2013) using *align.seqs*. Sequences were removed if they contained homopolymers longer than 8 bp or did not align to the correct region in the SILVA SEED alignment reference using *screen.seqs*. To further de-noise the data, sequences were pre-clustered within each sample allowing a maximum of 2 base pair differences between sequences using *pre.cluster.* Finally, chimeric sequences were removed using UCHIME (Edgar et al., 2011).

Quality filtered sequences were grouped into operational taxonomic units (OTUs) based on 97% sequence identity and classified using the Bayesian classifier and the Greengenes database (August 2013 release of gg_13_8_99) (DeSantis et al., 2006) with *classify.seqs*. Sequences that classified as mitochondria, chloroplasts, eukaryotes, or that were of unknown origin were removed using *remove.lineage.* To ensure normalization of data across samples, samples were rarefied to 10,340 sequences per sample, the smallest number of sequences across all collected samples (**Supplementary Figure S2**). After pooling samples by sampling site, singleton and doubleton abundances were calculated with *filter.shared.* Chao1 diversity indices (Chao, 1984), Good’s coverage (Good, 1953), Shannon indices (Shannon, 1948), and inverse Simpson indices were calculated using *summary.single* to quantify coverage and alpha diversity for individual and pooled samples. Beta diversity, the dissimilarity among community structures of each sample, was quantified in a 𝜃_Y C_ distance matrix (Yue and Clayton, 2005) using *dist.shared*, which was used for ordination analysis in a two-dimensional non-metric dimensional scaling (NMDS) plot (**Supplementary Figure S3**). Analysis of molecular variance (AMOVA) (Excoffier et al., 1992) was used to identify significant differences in community structure across the three locations using the 𝜃YC distance matrix for the *amova* command, while linear discriminant analysis (LDA) effect size (LEfSe) (Segata et al., 2011) was used to identify indicator taxa that were significantly enriched in their respective samples.

PICRUSt (Langille et al., 2013) was used to predict N cycling functionality using the metagenome_contributions.py script and the -l option to specify KEGG orthologs in the output for N fixation (K02588, K02586, K02591, K00531), nitrification (K10944, K10945, K10946, K10535), denitrification (K00368, K15864, K04561, K02305, K00376), dissimilatory nitrate reduction (K00370, K00371, K00374, K00373, K02567, K02568, K00362, K00363, K03385, K15876), assimilatory nitrate reduction (K00367, K10534, K00372, K00360, K00366, K17877), nitrate/nitrite transport (K02575), and ammonia assimilation (K00264, K00265, K00266, K01915, K01948).

### Statistical Analysis

One-way analysis of variance (ANOVA) was used to detect differences among the three experimental groups (LAG, INF, and EFF) for diversity indices and gene counts of N-cycling pathways using SigmaPlot (SigmaPlot 11.0, Systat Software, Inc., San Jose, CA, United States). For significant differences, *post hoc* Holm-Sidak multiple pairwise comparisons were used to detect differences between each pair of experimental groups. Significant differences were defined as *p <* 0.05.

### Emissions Measurements

Gas concentrations of ammonia (NH_3_), nitrous oxide (N_2_O), carbon dioxide (CO_2_), and methane (CH_4_) were measured over 24 h periods from the LAG, INF, EFF, surface (TOP) and the bottom gravel layer (BOT). Emission from TOP and BOT were measured directly from the vermiltration system while operating. For each of the wastewaters (i.e., LAG, INF, and EFF), gaseous emissions were measured in 20 L flux chambers containing 5 L sample of the specific wastewater using an inlet sampling the headspace above the wastewater. The LAG sample was collected from a pipe on the solids separator discharging water from the surface of the lagoon. The INF sample was collected from a spigot between the INF storage tank and the vermifilter. The EFF was collected from the pipe discharging EFF into the EFF storage tank. Emissions from the TOP of the vermifilter were captured using a triangular sampling tunnel covering a section of the surface of the vermifilter using an inlet affixed to the inside of the sampling tunnel (**Supplementary Figure S1**). Emissions from the BOT of the vermifilter were sampled using an inlet threaded through an exhaust pipe extending to the bottom of the vermifilter (**Supplementary Figure S1**). All air sampled at inlets were measured for NH_3_, N_2_O, CO_2_, and CH_4_ using the Mobile Agricultural Air Quality (MAAQ) laboratory as described previously (Mitloehner, 2016). Gas concentrations were used to calculate emission rates at each sampling site and the daily emissions rates of the 50,000 L system were subsequently used to calculate the daily gas production of the vermifilter.

### Wastewater Chemistry Analysis

Wastewater chemistry analyses of INF and EFF wastewater were performed at Denele Analytical, Inc. (Turlock, CA, United States) using standard analytical methods as described previously (Kopp and McKee, 1979; APHA, 2005).

## Results

### Sequencing and Quality Filtering

A total of 188,870 reads were generated from the three sampling sites (LAG, INF, and EFF), with a mean of 62,957 reads per sampling site. Quality filtering removed 39,567 (20.9%) of the generated reads and the remaining 149,303 reads were pooled by location and then assigned to 4,732 OTUs at 97% sequence identity (**Table 1**). Of the 4,732 OTUs, 1,551 (32.8%) were found in LAG, 1,157 (24.5%) in INF, and 3,349 (70.8%) in EFF (**Table 1**). The relative portion of singleton and doubletons varied among the three locations, with samples from INF and EFF having the lowest and highest portions of both singletons and doubletons respectively. More specifically, after quality filtering, the LAG, INF, and EFF contained 653 (1.1% of quality-filtered reads), 396 (0.7%), and 1,516 (4.6%) singletons, and 220 (0.4%), 155 (0.3%), and 546 (1.7%) doubletons, respectively (**Table 1**).

**Table 1:** Sequence count, OTU richness, and coverage of prokaryotic 16S rRNA gene sequences of biological replicates (*n* = 3) pooled according to location.

	LAG	INF	EFF	Total count
Raw reads	72,756 (100%)	73,611 (100%)	42,503 (100%)	188,870 (100%)
Quality filtered reads	57,466 (79.0%)	58,977 (80.1%)	32,860 (77*3%)*	149,303 (79.0%)
Doubletons	220	155	546	
Singletons	653	396	1,516	
Observed OTUs	1,551	1,157	3,349	4, 732
OTUs, excluding singletons	898	761	1,833	
OTUs, excluding singletons and doubletons	678	606	1,287	
Good’s coverage [%]	96.9	97.7	94.4	

### Alpha Diversity

To estimate the microbial diversity within each sample, species richness estimators, and community diversity indices were calculated and averaged across triplicates (**Table 2**) and rarefaction analyses were performed. Differences in the Chao 1 estimates and the inverse Simpson index between EFF vs. LAG and EFF vs. INF were not significant (ANOVA *p* > 0.05), and since these tests had <80% power, it is possible that significant differences may have been detected with a greater number of samples. Furthermore, it is possible that an increased proportion of solid in LAG and INF caused increased heterogeneity and subsequently resulted in higher standard deviation of the Chao1 from the LAG and INF compared to the more homogenous EFF. Rarefaction curves (**Supplementary Figure S2**) indicated that the EFF had the highest number of OTUs, which was in accordance with the estimated OTU counts. Good’s coverage estimates of 94.4, 96.9, and 97.7% were calculated for EFF, LAG in INF, respectively (**Table 1**) indicating that sequencing efforts were sufficient to recover a large fraction of the microbial population in each of the samples. Both the Shannon diversity index and the non-parametric Shannon index were significantly higher in the EFF microbial community compared to the LAG (Holm-Sidak test, *p =* 0.001) and the INF (Holm-Sidak test, *p =* 0.001) communities, suggesting that the EFF harbored not only more different OTUs, but also a considerable abundance of these different OTUs (**Table 2**).

**Table 2:** Diversity indices of prokaryotic 16S rRNA gene sequences averaged over replicates (*n =* 3).

	Mean (*SD*)		
	LAG	INF	EFF
Chao1	2264 (1365)	1553 (803)	3062 (75)
Shannon	2.4 (0.5)	2.1 (0.3)	4.8 (0.5)
Non-parametric Shannon	2.5 (0.6)	2.2 (0.3)	4.9 (0.4)
Inverse Simpson	2.3 (0.4)	2.1 (0.2)	8.4 (2.6)

*SD indicates standard deviation.*

### Beta Diversity

Variance of the community structure among the wastewater samples was quantified using a 𝜃_Y C_ distance matrix (Yue and Clayton, 2005) and subsequent AMOVA indicated that the genetic diversity within the three microbiomes was significantly different from that of their pooled genetic diversity (*p* = 0.017), with the LAG and INF microbiomes clustering closer to each other than to the EFF microbiome (**Supplementary Figure S3**). This clustering pattern was expected because the LAG and INF differed only by solids removal, whereas the EFF was the product of vermifiltration treatment. As a result, the EFF microbiome contained more unique OTUs than the LAG or INF microbiomes. Of the 4,732 OTUs identified across all samples, more than half of them (54.7%) were found in EFF exclusively, whereas LAG and INF contained substantially fewer exclusive OTUs (788 OTUs, 16.7%; 458 OTUs, 9.7% of all OTUs, respectively). Of the 4,732 OTUs, 427 (9%) core OTUs were shared among all three microbiomes, 564 (11.9%) between LAG and INF, 562 (11.9%) between INF and EFF, and 626 (13.2%) between LAG and EFF. A summary of the core OTUs across the different habitats is provided in **Supplementary Table S2**.

### Microbial Community Structure

**FIGURE 2:**
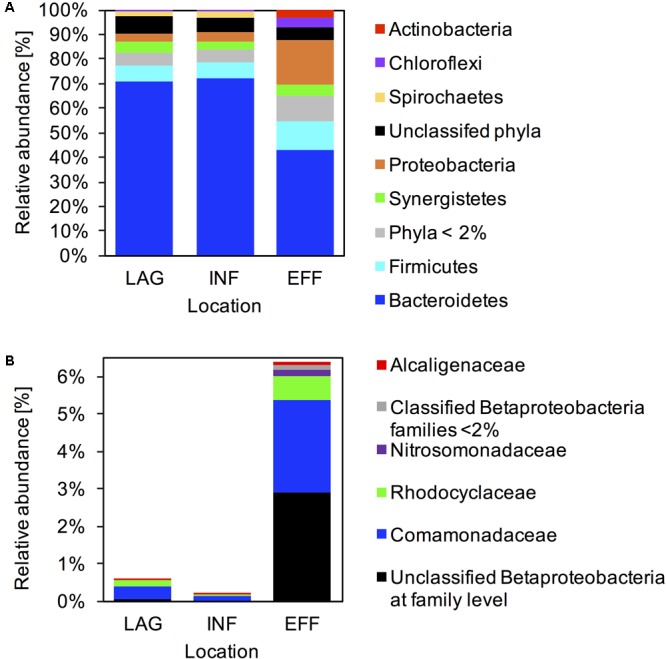
Microbial community composition. Relative abundance of 16S rRNA reads assigned to each taxon averaged across LAG, INF, and EFF wastewater samples at **(A)** the phyla level, and **(B)** within *Betaproteobacteria* at the family level. Taxonomy was assigned using Greengenes at 97% identity.

Across all samples, 4,732 OTUs were classified to 3 archaeal and 48 bacterial phyla. The 10 most abundant phyla recruited > 98% of the reads generated from the microbial communities in LAG and INF, whereas in the EFF, the 10 most abundant phyla recruited 93% of the generated reads (**Supplementary Table S3**). *Bacteroidetes* dominated the microbial communities associated with all three locations and were ∼1.6 times more abundant in LAG (70.6%) and INF (72.3%) compared with EFF (43.4%) (**Figure 2A** and **Supplementary Table S3**). In LAG and INF microbial communities, *Firmicutes* was the second most dominant phylum (7 and 6.1%, respectively), followed by *Synergistetes* (4.3 and 3.6%), *Proteobacteria* (3.8 and 3.3%), and *Spirochaetes* (1.7 and 2.3%). Contrarily, in EFF, *Proteobacteria* (18.1%) was the second most abundant phylum, and included *Betaproteobacteria* (6.4%), *Gammaproteobacteria* (6.3%), *Alphaproteobacteria* (3.3%), and *Deltaproteobacteria* (1.7%). Relative to the INF, the increase in *Betaproteobacteria* (29.5-fold) in the EFF was largely driven by an increase in *Comamonadaceae* (22.7-fold) and *Rhodocyclaceae* (10.2-fold), families within *Betaproteobacteria* (**Figure 2B** and **Supplementary Table S4**). After *Betaproteobacteria*, the third most abundant phylum in EFF was *Firmicutes* (11.3%). Relative to the INF, the EFF community contained a higher abundance of *Chloroflexi* (6.2-fold, 3.7%) and *Actinobacteria* (13.2-fold, 3.3%) and a lower abundance of *Bacteroidetes* (1.7-fold), *Spirochaetes* (5-fold, 0.4%), and members of the candidate phylum WWE1 (2.9-fold, 0.6%) (**Figure 2A** and **Supplementary Table S3**).

### Indicator Taxa

Overall, a total of 274 taxa at various phylogenic levels were highly abundant in their respective samples (LDA scores > 2.0). To focus on the most highly enriched taxa, only taxa with LDA scores > 3.5 (**Supplementary Figures S4, S5**) are highlighted in this text. The LAG microbiome contained a single classified taxon, an archaeon belonging to the order *Methanosarcinales* (from family to order), that was significantly enriched. The microbiome associated with INF was enriched in eight classified taxa distributed between two lineages: the candidate order PL_11B10 (from phylum to order) and bacteria belonging to the genera W22 (from phylum to genus). The EFF microbial community was enriched in the 15 classified taxa distributed among six lineages: *Acidimicrobiales* (from class to order), *Flavobacteriaceae* (from class to family), *Anaerolinaceae* (from order to family), bacteria belonging to the class *Turicibacter* (from order to genus), bacteria belonging to *Butyrbivrio* (order to genus), and *Comamonadaceae* (from class to family). Of these lineages, six had LDA scores of ≥ 4: in the INF, the order PL_11B10 and class MVP_22 both members of the *Spirochaetes*; and in the EFF, *Acidimicrobiales, Flavobacteriaceae, Betaproteobacteria*, and *Comamonadaceae*.

### Functional Gene Prediction

**FIGURE 3:**
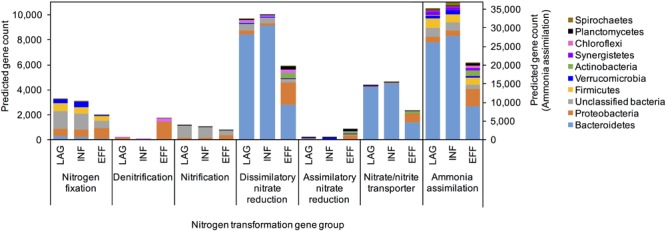
PICRUSt predicted average gene counts within each nitrogen transformation pathway from the top ten phyla with the most abundant nitrogen cycling gene counts.

**FIGURE 4:**
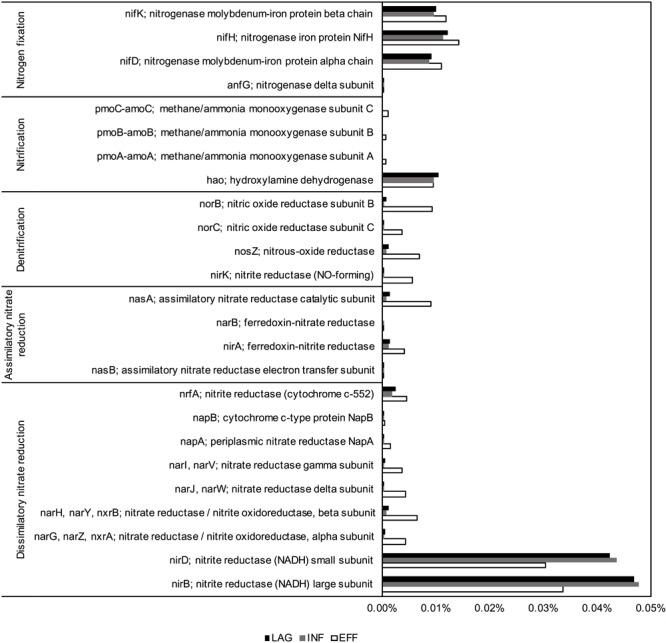
Relative abundance of nitrogen-cycling genes, as predicted by PICRUSt.

PICRUSt predicted that the EFF metagenome was significantly enriched in genes associated with denitrification and assimilatory nitrate reduction compared to the genes predicted within the metagenome of LAG (Holm-Sidak test, *p* = 0.009, *p* = 0.013, respectively) and INF (Holm-Sidak test, *p* = 0.009, *p* = 0.013, respectively). Members of the *Betaproteobacteria* were identified as primary contributors to the increased presence of genes involved in denitrification (**Figure 3**). ANOVA indicated that the predicted gene count for nitrate and nitrite transporter differed among the three microbiomes (*p* = 0.043). For N fixation and dissimilatory nitrate reduction, ANOVA for the overall sum of predicted genes within each of these pathways did not have sufficient power to confidently detect differences among the three wastewaters. However, upon closer examination of the phyla that contributed to these two pathways, the community composition of the EFF metagenome differed from that of the LAG and INF (**Figure 3**). For nitrification, no significant difference was detected for the sum of all genes associated with this pathway (ANOVA, *p* > 0.05); however, among the genes associated with nitrification, only the EFF was predicted to have genes encoding ammonia monooxygenase (*amoC, amoB*, and *amoA*), while abundances for hydroxylamine oxygenase (*hao*) were similar across all three microbiomes (**Figure 4**). Correspondingly, only the EFF metagenome was predicted to have all genes associated with the entire N cycle.

### Gaseous Emission and Water Chemistry Data

Vermifiltration reduced emission of ammonia from EFF by 90.2% compared to the INF without significantly increasing emission of N_2_O, CH_4_, or CO_2_ (**Table 3**). The water chemistry analysis revealed a 2-fold reduction in total Kjeldahl N and a 3-fold reduction in NH_3_ from the INF to the EFF coupled with only a slight increase in NO_3_^-^ and NO_2_^-^ (**Table 4**).

**Table 3:** Daily net emissions from the wastewaters (LAG, INF, and EFF), the TOP and BOT of the vermifilter, and the vermifilter itself.

	Daily net emission						Gas removal efficiency of the vermifilter^∗^ [%]
	Q_lag_	Q_inf_	Q_eff_	Q_top_	Q_bot_	Q_V ermifilter_	
NH_3_ [kg d^-1^]	17.6	17.2	1.5	0.1	0.0	15.5	90.2%
N_2_O [kg d^-1^]	2.3E-04	1.7E-03	1.3E-03	1.3E-01	1.3E-02	–1.5E-01	–8,685.1%
CO_2_ [kg d^-1^]	75.0	97.1	43.7	54.5	4.9	–6.0	–6.1%
CH_4_ [kg d^-1^]	0.4	0.6	0.3	0.8	0.0	–0.5	–84.4%

*^∗^Negative values indicate that the vermifilter contributed emissions to the wastewater.*

**Table 4:** Wastewater chemistry analysis of the influent and effluent from the vermifilter.

		Sample location	
Constituent	Units	INF	EFF
Ammonia	mM	11.69	3.78
Electrical conductivity	mmhos/cm	6.29	6.70
Nitrate	mM	^∗^	2.46
Nitrite	mM	^∗^	0.18
pH		7.60	7.76
Soluble salts	ppm	4,026	4288
Total Kjeldahl Nitrogen	mM	19.20	8.42

*^∗^Below limit of detection.*

## Discussion

In the study presented here, we used Illumina amplicon sequencing to assess the dynamics of the microbiome composition during vermifiltration of dairy wastewater and whether changes in the community composition reflected an enhanced N removal potential. The obtained results supported our hypothesis and demonstrated that vermifiltration shifted the microbial community structure by enriching the wastewater microbiome in taxa known to participate in nutrient degradation and cycling. Further functional gene analysis of the microbiomes suggested vermifiltration enriched the wastewater in bacteria and archaea capable of N transformations. The enhanced N-cycling capability of the microbial community of the treated wastewater echoes the conclusions drawn from the water chemistry analysis and the NH_3_ and N_2_O emission profiles. The reduction in soluble and volatile NH_3_ and accumulation of soluble NO_3_^-^ and NO_2_^-^ are signatures of ongoing nitrification. Although daily N_2_O net emission from the vermifilter was ∼87-fold greater than N_2_O emission measured from the influent (INF), overall net emission of N_2_O, with greatest amounts of N_2_O being released from the top section (TOP) of the vermifiltration system, were minuscule (around the limit of quantification). Minute emission of N_2_O despite the overall reduction in NH_3_ and total Kjeldahl N indicate complete denitrification to N_2_ as opposed to incomplete denitrification to N_2_O (**Tables 3, 4**). Taking the overall reduction of nitrogen, in the emitted gasses and in the dairy wastewater, into consideration it our data suggest that vermifiltration is a sustainable approach to reduce the nitrogen load of dairy wastewater.

The shift in microbiota was prevalent at the phyla level, with *Proteobacteria* becoming more abundant and *Bacteroidetes* becoming less abundant through the vermifiltration process (**Figure 2A**). *Bacteroidetes* were initially highly abundant in the INF microbiome, and continued to be the most abundant phylogenetic group in the effluent, although the abundance of this phylum decreased from 72.3% (INF) to 43.4% (EFF). Vermifiltration also enriched the EFF in *Proteobacteria*, mainly by increasing the abundance of members belonging to *Betaproteobacteria*. High abundance of *Proteobacteria* in the EFF microbiome was in concordance with previous studies that reported *Proteobacteria* as the most highly abundant phyla in the biofilm coating the media within the vermifilter. In these studies, the most abundant families within *Proteobacteria* were the *Betaproteobacteria* and *Gammaproteobacteria* (Zhao et al., 2010; Liu et al., 2012, 2013; Li et al., 2013; Xu et al., 2014), which include ammonia-oxidizing (Koops et al., 2006) and denitrifying bacteria (Thomsen et al., 2007; McIlroy et al., 2016). It is not clear whether the increased abundance of *Proteobacteria* in the EFF is due to an enhanced growth of these taxa in the wastewater, or that these microorganisms were dislodged from the biofilm as the wastewater percolated through the media and thus represent washout from the biofilm of the vermifilter. We hypothesize that the observed changes are most likely due to a combination of these events, and that many of the detected microorganisms were actively metabolizing within the effluent.

AMOVA indicated the community structure among all three locations differed from each other, with the LAG and INF communities more similar each other than to the EFF community, and the individual EFF replicates exhibiting greater variance than the replicates of LAG or INF. This suggests that removal of solid particles from the wastewater stream prior to applying the water to the vermifilter was too transient to elicit a detectable effect on the taxonomic assemblage of the microbial community. As a result, LEfSe identified the most discriminatory taxa at a finer scale for the EFF microbiome compared with the microbiomes of the LAG and INF (**Supplementary Figures S4, S5** and **Supplementary Table S5**). Comparing the functions of these taxa suggested that vermifiltration shifted the metabolism of the discriminatory taxa from methanogens to microorganisms responsible for N transformations and decomposition. LAG was enriched in *Methanosarcinales*, an order containing methanogens previously detected in anaerobic digesters (Friedrich, 2005; Ellis et al., 2012; Maspolim et al., 2015). INF was enriched in *Cloacamonaceae*, a family belonging to the candidate division WWE1 that includes *Candidatus Cloacamonas acidaminovorans*. *Candidatus Cloacamonas acidaminovorans* is an anaerobic bacterium found previously in anaerobic digesters (Siezen and Galardini, 2008; Muller et al., 2016) and is hypothesized to be a syntroph capable of fermenting amino acids to produce energy and carbon (Pelletier et al., 2008). In the EFF microbiome, the enrichment of *Betaproteobacteria* was largely driven by the increased abundance of *Comamonadaceae*, a family previously known to represent dominant denitrifiers during wastewater treatment (Adav et al., 2009; McIlroy et al., 2016; Wang and Chu, 2016). Similarly, the enrichment of *Comamonadaceae* in the EFF suggests that members of this family are responsible for denitrification during the vermifiltration process. The EFF microbiome was also enriched in *Flavobacteriaceae*, a family containing mostly anaerobes and microanaerobes known to decompose organic material and participate in biogeochemical cycling (Bernardet and Nakagawa, 2006). Members of *Flavobacteriaceae* have been previously reported to occur in anaerobic digesters (De Vrieze et al., 2015; Maspolim et al., 2015), which supports previous work that indicated that the vermifiltration process is characterized by the presence of aerobic and anaerobic microhabitats.

In addition to assessing the function of indicator taxa, we performed a comparative analysis of the N cycling potential of the microbiomes from the three different sampling locations using the predicted gene counts for each N transformation pathway (i.e., N fixation, denitrification, nitrification, dissimilatory nitrate reduction, assimilatory nitrate reduction, nitrate/nitrite transporter, NH_3_ assimilation). Because the majority of N in the dairy wastewater is in the form of NH_3_ from dairy slurry, the NH_3_-rich wastewater is poised to enter the N cycle at the nitrification step. At the pathway scale, all three microbiomes had similar predicted counts of genes associated with N fixation and nitrification at the pathway scale. However, at the individual gene level within each pathway, only the EFF contained the *amo* genes encoding ammonia monooxygenase, which catalyzes the first step of nitrification, converting NH_3_ to hydroxylamine (NH_2_OH) (**Supplementary Figure S6**). In addition to possessing the necessary *amo* genes, the EFF metagenome was enriched in denitrification genes compared to the metagenomes of LAG and INF, including *nosZ*, which catalyzes the last step of denitrification from N_2_O to atmospheric N_2_. Complete denitrification to N_2_ is ideal because N_2_O, a byproduct of incomplete denitrification, is a greenhouse gas 298 times more potent than N_2_ (IPCC, 2013). Effectively, only the EFF microbiome possessed all the genes necessary for every step of the N cycle, enabling the microbial community to convert NH_3_ completely to the benign gas N_2_ (**Supplementary Figure S6**). Accordingly, the nitrogenous gas emission profiles from EFF indicated a ∼90% reduction of NH_3_ emission without a significant increase in N_2_O emission (**Table 3**). Although the LAG and INF microbiomes had an equivalent gene count across all nitrification genes, these two microbiomes theoretically could not carry out nitrification due to the absence of ammonia monooxygenase. This would result in a lack of substrate for subsequent denitrification, which in turn would hamper the growth of denitrifiers and consequentially explain the lower counts of denitrification genes observed for the LAG and INF microbiomes (**Figures 3, 4**).

Metabolic interference has been used widely to predict the metabolic function and the presence of functional key enzymes. Although results from this phylogeny-based approach should only be considered as a preliminary indicator that these metabolic pathways are likely to be present, prediction accuracy of PICRUSt has been shown to be rather high (∼98%) for nitrogen metabolism in general (Langille et al., 2013). Hence it cannot be excluded that there are differences in the accuracy with which individual gene and pathway abundances are predicted using this approach, but the clear benefit of this in-silico technique remains its capability to outperform shallow metagenomic sequencing in predicting the presence and absence of specific functions of interest (Langille et al., 2013). It therefore provides a valuable tool to obtain initial insights and the opportunity to make informed decisions when planning more in-depth studies. It is also important to keep in mind that comparative analyzes based on the abundance of different genes and pathways have to be performed with great caution since the ability to detect particular genes and their associated functions depends heavily on the reference database and the primer set used for amplification of the phylogenetic marker region. For example, the overall abundance of the nitrogen fixation genes observed throughout the vermifilter appears to exceed the abundance of some of the other genes involve in nitrogen utilization. Taking the previously mentioned shortcomings of metabolic interference into consideration, it would be ill advice to conclude from these data that the nitrogen fixing population is highly active during vermifiltration; however, it appears safe to conclude that this population is present throughout vermifiltration process at similar abundance. Its metabolic activity is currently unknown and more targeted and holistic approaches will be required for an enhanced understanding of its contribution to the N-cycling process during vermifiltration. Follow-up experiments utilizing shotgun metagenomics, metatranscriptomics or RT-PCR should be considered, although these approaches have their specific challenges as well (i.e., high costs and lack of specific RT-PCR primers targeting the functional genes present in the environmental sample).

Although vermifiltration is known to increase the overall aeration of the wastewater, our results suggest that the vermifilter contains aerobic and anaerobic microhabitats that allow the enrichment of microorganisms containing genes necessary for complete conversion of organic N into gaseous N_2_. The dual (aerobic/anaerobic) nature of the vermifilter provides an ideal ecosystem to facilitate the microbial decomposition and removal of organic N from dairy wastewater, decreasing the downstream N load on the soil or groundwater without increasing gaseous emissions that expedite climate change. While it is still unclear which specific organisms contribute to the N-removing processes and what their spatial distribution is within the vermifilter, results from this study provide valuable insights into the microbiology of the vermifiltration process at an unprecedented level of resolution. Furthermore, the findings presented here establish a framework to design strategies for optimizing N-removal via vermifiltration, a technology that holds much promise for improving the sustainability of waste management for the livestock industry. Before these strategies can be developed, it would be beneficial to expand our work and evaluate whether results can be reproduced using more sample replicates across multiple dairies over several seasons. In addition, metagenomics shotgun sequencing as well as gene expression or protein profiling of the microbiomes would more accurately assay the molecular processes that occur during the treatment process of dairy wastewater in the storage lagoon and during vermifiltration.

## Author Contributions

All authors listed have made a substantial, direct and intellectual contribution to the work, and approved it for publication.

## Conflict of Interest Statement

The authors declare that the research was conducted in the absence of any commercial or financial relationships that could be construed as a potential conflict of interest.

## Supplementary Material

FIGURE S1 Vermifilter design and emission sampling sites. The vermifilter is a concrete enclosure (49 m × 11 m × 1.5 m) filled woodchips inoculated with earthworms and microorganisms to enhance solids and contaminant removal. The large particle size of the woodchips, the bottom layer of gravel, and the exhaust pipes that line the perimeter of the vermifilter enhance aeration of the vermifilter. After the lagoon water (LAG) has passed through the solids separator, lawn sprinklers apply the resulting influent (INF) over the surface (TOP) of the vermifilter. The INF filters to the bottom (BOT) of the vermifilter, resulting in the effluent (EFF). Gas samples from wastewater were collected from flux chambers for the wastewaters (LAG, INF, and EFF), a triangle sampling tunnel for the TOP, and tubing threaded to the bottom of an exhaust pipe for the BOT. Diamond arrow indicate where inlets for gas sampling were placed. All gas samples were transferred via tubing to the Mobile Agricultural Air Quality Lab, where gas analyzers measured the amounts of NH_3_, N_2_O, CO_2_, and CH_4_. Figure not to scale.

FIGURE S2 Rarefaction curves. Rarefaction analysis of the V4 region of the 16S rRNA gene from three replicates (A, B and C) of three dairy wastewater samples (LAG, lagoon water; INF, influent; and EFF, effluent) constructed at 97% sequence similarity.

FIGURE S3 Non-metric dimensional (NMDS) scaling ordination analysis of dairy wastewater samples from different locations in the vermifiltration system. Each point corresponds to one triplicate from each of the three locations: lagoon water (LAG), influent (INF), and effluent (EFF).

FIGURE S4 Significantly enriched classified taxa characterizing LAG, INF, and EFF with LDA scores > 3.5, as determined by LEfSe.

FIGURE S5 Cladogram depicting the phylogenetic distribution of significantly enriched microbial taxa as determined by LEfSe in the three types of dairy wastewater samples: lagoon water (LAG), influent (INF), and effluent (EFF). Each circle represents a taxa level, from the inner circle representing phyla to the outer circle representing species. Each dot represents a clade and is colored according to which samples in which they are significantly abundant, as determined by LEfSe with LDA scores > 3.5: blue for LAG, green for INF, red for EFF, and yellow for non-discriminate. The diameter of each dot is proportional to the taxa’s relative abundance. Labeled, highlighted wedges indicate discriminatory phyla, classes, orders, families, and genera.

FIGURE S6 The nitrogen cycle in dairy production. Dinitrogen (N_2_) is fixed into ammonium (NH_4_^+^), which undergoes nitrification to produce nitrite (NO_2_^-^) and nitrate (NO_3_^-^). Crops use NH_4_^+^, NO_2_^-^, and NO_3_^-^ to make proteins. These crops are then harvested and fed to dairy cattle, who use the plant proteins to make their own proteins. Dairy cattle excrete nitrogenous waste as urea [CO(NH_2_)_2_] in their urine. Upon contact between urine and feces, urease in the feces hydrolyzes CO(NH_2_)_2_ from the urine to NH_4_^+^, which undergoes nitrification to NO_2_^-^, and NO_3_^-^. Finally, complete denitrification converts NO_3_^-^ first to N_2_O and finally to N_2_, whereas incomplete denitrification fails to progress beyond N_2_O.

TABLE S1 Barcode sequences used in this study.

TABLE S2 Core OTUs shared among all three wastewater samples (lagoon water, LAG; influent, INF; and effluent, EFF). OTUs classified at the genus level are listed first, followed by unclassified OTUs. Because some OTUs share the same taxa classification (i.e., 97% sequence identity defines an OTU, but multiple OTUs are classified to or unclassified at the same lineage), the count of OTUs with the same taxa classification (*n*) is also listed.

TABLE S3 Community composition of dairy wastewater samples during different stages of vermifiltration at the phylum level averaged over replicates (*n =* 3).

TABLE S4 Community composition of *Betaproteobacteria* at the family level in dairy wastewater samples during different stages of vermifiltration averaged over replicates (*n =* 3).

TABLE S5 Significantly enriched taxa with LDA scores > 3.3. Clades within each lineage are listed from kingdom to species, and OTU, separated by commas. Unclassified clades share the same label as the clade at which they are unclassified.
