# Supplementary figures and images for: Profiling of the Microbiome Associated With Nitrogen Removal During Vermifiltration of Wastewater From a Commercial Dairy
Source: Front Microbiol. 2018 Aug 20;9:1964. doi: 10.3389/fmicb.2018.01964 (PMC6110276; doi:10.3389/fmicb.2018.01964)

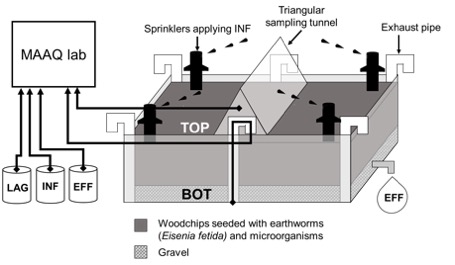

Supplement: FIGURE S1 — Vermifilter design and emission sampling sites. The vermifilter is a concrete enclosure (49 m × 11 m × 1.5 m) filled woodchips inoculated with earthworms and microorganisms to enhance solids and contaminant removal. The large particle size of the woodchips, the bottom layer of gravel, and the exhaust pipes that line the perimeter of the vermifilter enhance aeration of the vermifilter. After the lagoon water (LAG) has passed through the solids separator, lawn sprinklers apply the resulting influent (INF) over the surface (TOP) of the vermifilter. The INF filters to the bottom (BOT) of the vermifilter, resulting in the effluent (EFF). Gas samples from wastewater were collected from flux chambers for the wastewaters (LAG, INF, and EFF), a triangle sampling tunnel for the TOP, and tubing threaded to the bottom of an exhaust pipe for the BOT. Diamond arrow indicate where inlets for gas sampling were placed. All gas samples were transferred via tubing to the Mobile Agricultural Air Quality Lab, where gas analyzers measured the amounts of NH3, N2O, CO2, and CH4. Figure not to scale. [file Image_1.TIFF]

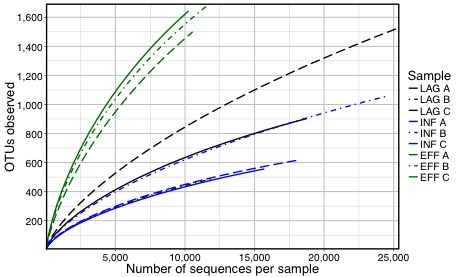

Supplement: FIGURE S2 — Rarefaction curves. Rarefaction analysis of the V4 region of the 16S rRNA gene from three replicates (A, B and C) of three dairy wastewater samples (LAG, lagoon water; INF, influent; and EFF, effluent) constructed at 97% sequence similarity. [file Image_2.TIFF]

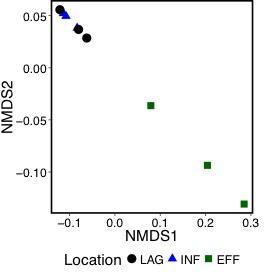

Supplement: FIGURE S3 — Non-metric dimensional (NMDS) scaling ordination analysis of dairy wastewater samples from different locations in the vermifiltration system. Each point corresponds to one triplicate from each of the three locations: lagoon water (LAG), influent (INF), and effluent (EFF). [file Image_3.TIFF]

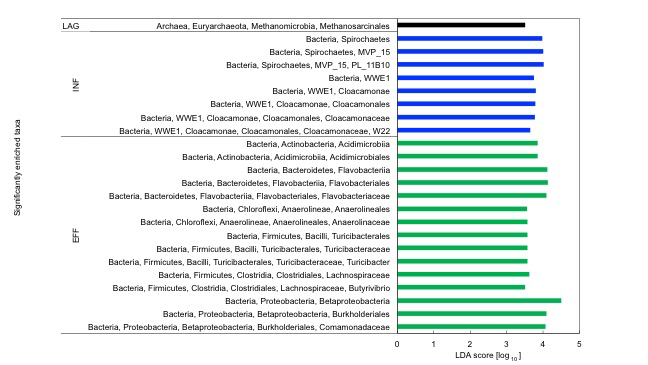

Supplement: FIGURE S4 — Significantly enriched classified taxa characterizing LAG, INF, and EFF with LDA scores > 3.5, as determined by LEfSe. [file Image_4.TIFF]

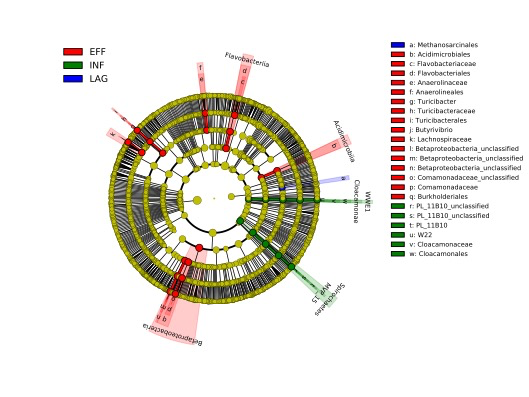

Supplement: FIGURE S5 — Cladogram depicting the phylogenetic distribution of significantly enriched microbial taxa as determined by LEfSe in the three types of dairy wastewater samples: lagoon water (LAG), influent (INF), and effluent (EFF). Each circle represents a taxa level, from the inner circle representing phyla to the outer circle representing species. Each dot represents a clade and is colored according to which samples in which they are significantly abundant, as determined by LEfSe with LDA scores > 3.5: blue for LAG, green for INF, red for EFF, and yellow for non-discriminate. The diameter of each dot is proportional to the taxa’s relative abundance. Labeled, highlighted wedges indicate discriminatory phyla, classes, orders, families, and genera. [file Image_5.TIFF]

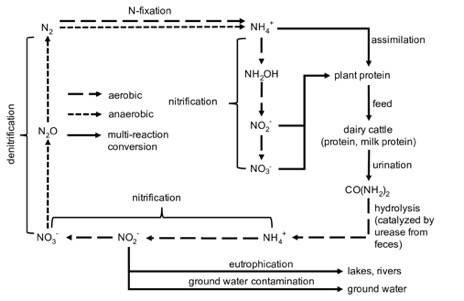

Supplement: FIGURE S6 — The nitrogen cycle in dairy production. Dinitrogen (N2) is fixed into ammonium (NH4+), which undergoes nitrification to produce nitrite (NO2-) and nitrate (NO3-). Crops use NH4+, NO2-, and NO3- to make proteins. These crops are then harvested and fed to dairy cattle, who use the plant proteins to make their own proteins. Dairy cattle excrete nitrogenous waste as urea [CO(NH2)2] in their urine. Upon contact between urine and feces, urease in the feces hydrolyzes CO(NH2)2 from the urine to NH4+, which undergoes nitrification to NO2-, and NO3-. Finally, complete denitrification converts NO3- first to N2O and finally to N2, whereas incomplete denitrification fails to progress beyond N2O. [file Image_6.TIFF]
